# Angina Simultaneously Diagnosed with the Recurrence of Myalgic Encephalomyelitis/Chronic Fatigue Syndrome

**DOI:** 10.3390/diagnostics11030460

**Published:** 2021-03-06

**Authors:** Koki Li, Yuki Otsuka, Yasuhiro Nakano, Daisuke Omura, Kou Hasegawa, Mikako Obika, Keigo Ueda, Hitomi Kataoka, Fumio Otsuka

**Affiliations:** 1 Department of General Medicine, Okayama University Graduate School of Medicine, Dentistry and Pharmaceutical Sciences, Okayama 700-8558, Japan; p94k6prv@s.okayama-u.ac.jp (K.L.); y-nakano@okayama-u.ac.jp (Y.N.); me20011@s.okayama-u.ac.jp (D.O.); khasegawa387@yahoo.co.jp (K.H.); obika-m@cc.okayama-u.ac.jp (M.O.); philopotchy@live.jp (K.U.); hitomik@md.okayama-u.ac.jp (H.K.); fumiotsu@md.okayama-u.ac.jp (F.O.); 2 Center for Graduate Medical Education, Okayama University Hospital, Okayama 700-8558, Japan

**Keywords:** chronic fatigue syndrome, endothelial dysfunction, microvascular angina, myalgic encephalomyelitis, vasospastic angina

## Abstract

Myalgic encephalomyelitis/chronic fatigue syndrome (ME/CFS) mainly affects young adults and can have a potential impact on social functioning. As this syndrome is associated with endothelial dysfunction, the heart can be damaged via ischemia due to endothelial damage. This might potentially lead to heart failure, which accounts for approximately 20% of deaths among patients with ME/CFS. While cardiac ischemia is thought be a pathophysiologically important manifestation of this syndrome, this is not yet reported. Herein, we present a case of a young female with newly diagnosed vasospastic or microvascular angina and concurrent exacerbation of ME/CFS severity. Her anginal symptoms, including exertional chest pain and transient chest discomfort, mimicked those of ME/CFS but were relieved after the administration of a calcium channel blocker. We emphasize the possibility of concurrent angina and exacerbation of ME/CFS and the importance of detecting cardiac ischemia to avoid unfavorable outcomes.

## 1. Introduction

Myalgic encephalomyelitis/chronic fatigue syndrome (ME/CFS) is characterized by extreme fatigue that lasts at least 6 months, and post-exertional malaise [1]. There are several diagnostic criteria like the Fukuda Case definition for CFS [1], Canadian Consensus Criteria for ME/CFS [2], and the Institute of Medicine 2015 Diagnostic Criteria [3]. For a diagnosis to be made, some associated symptoms such as autonomic symptoms (e.g., orthostatic hypotension), neurocognitive symptoms (e.g., impaired memory and attention deficit), immune symptoms (e.g., lymphadenopathy, sore throat, and fever), and other symptoms (e.g., sleep disturbance, headache, muscle pain, and multi-joint pain) are often required, along with ruling out the presence of any medical conditions that could explain the presence of fatigue.

In terms of epidemiology, more than 1% of the population might be affected by ME/CFS, which preferentially affects the younger female population. Prior studies conducted in Norway reported two peaks for the age of onset—10–19 and 30–39 years [4,5]. 

While it is well-established that ME/CFS imposes a profound burden on society, its etiology and pathophysiology are not yet fully understood. Endothelial dysfunction is a pathological manifestation that is associated with this disease, and it plays a major role in the development of angina and other coronary diseases [6]. Previous studies also suggest that ME/CFS is a potential cause of cardiac ischemia [7,8]. Lerner et al. reported repeated negative T-wave changes among patients with ME/CFS [9]. However, to our knowledge, no prior studies reported the new diagnosis of coronary disease in patients with preexisting ME/CFS. Herein, we report a case of ME/CFS in which microvascular angina or vasospastic angina with an electrocardiogram (ECG) change was newly diagnosed in a patient with concurrent exacerbation of ME/CFS. This report highlights the importance of cardiac investigation for obtaining a better insight into this condition and assessing subsequent patient prognosis.

## 2. Case Presentation

A 23-year-old single woman, who was previously healthy and used to play football, was referred and admitted to our department for the investigation of chronic fatigue, which persisted for 9 months. She first started to feel an excessive fatigue even when resting, which often prevented her from stepping out, 9 months earlier. She complained that daily activities such as shopping and housework were enough to deteriorate the fatigue. In addition to fatigue, she had insufficient sleep, mood disturbances, and difficulty in concentrating as neurocognitive symptoms; a slight fever as the only immune symptom; myalgia, multi-joint pain, and headache which does not respond to NSAIDs, dyspnea, and palpitations. There were no signs of sore throat or lymphadenopathy. The results of her ECG, chest X-ray, and blood tests (including liver function test, thyroid function test, and adrenal function test) were all within the normal ranges on admission. There were no signs of infection, organ failure, rheumatologic disease, or malignancy. A diagnosis of depression was ruled out by a psychiatrist. Schizophrenia, manic-depressive illness, substance abuse, eating disorder, and proven organic brain disease were also excluded. The 30-min head-up tilt test indicated that the patient had an orthostatic disorder as an autonomic manifestation. Meeting all of the Fukuda criteria, Canadian Consensus criteria for ME/CFS, and the Institute of Medicine criteria, she was eventually diagnosed with ME/CFS [1,2,3]. Her performance status (PS) (Table 1) was classified as 5, considering that she was not able to work or play football as usual [10]. Kampo medicine was subsequently prescribed, and her condition gradually improved. She recovered 9 months after admission and was able to resume work. She also married about 2 months after resuming work. However, the symptoms recurred 1 year after her initial admission, and her fatigue worsened. She experienced transient chest pain associated with relatively light labor, such as shopping; she did not initially seek treatment, as she thought that these symptoms were due to an exacerbation of ME/CFS.

**Figure 1:**
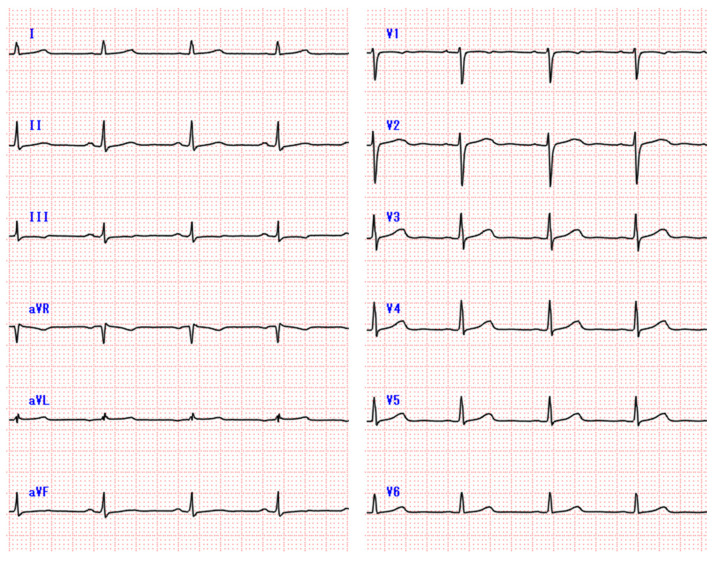
Resting electrocardiogram on second admission. No abnormal electrocardiogram changes were detected on admission.

**Figure 2:**
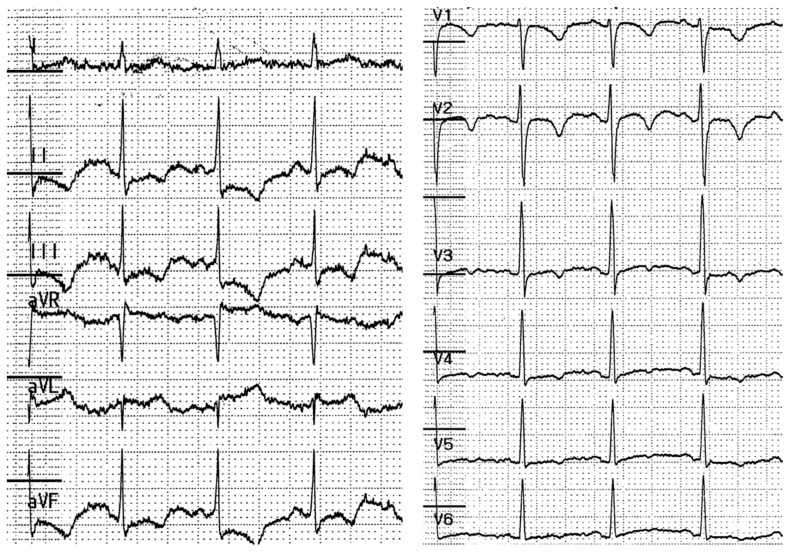
Electrocardiogram obtained when the patient complained of transient chest discomfort. ST depression and negative T waves were recorded in leads II, III, and aVF.

**Figure 3:**
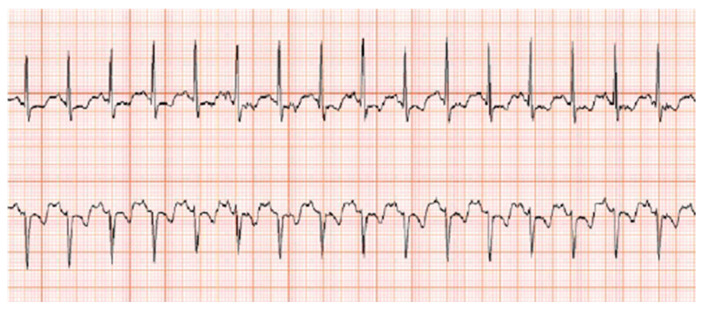
24-h holter monitoring. ST depression and negative T waves with tachycardia were recorded via 24-h holter monitoring; this was concurrent with the patient’s report of exacerbated fatigue.

**Figure 4:**
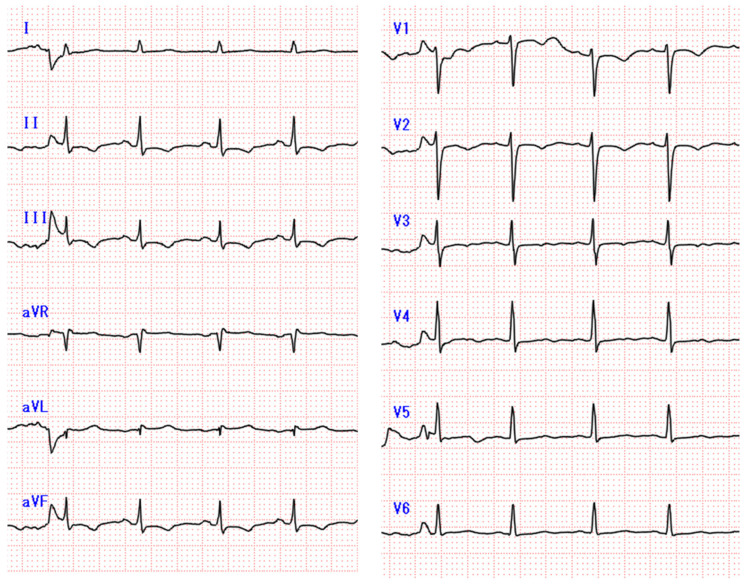
Two-step exercise electrocardiogram. Two-step exercise provoked wide ST depression and negative T waves on the electrocardiogram. I, II, III, aVL, aVR and aVF are each corresponding to six limb leads, and V1, V2, V3, V4, V5 and V6 are each corresponding to six precordial leads.

Her symptoms continued to worsen, and her activities of daily living were significantly impaired; for example, she reported being only able to eat a small portion of her regular meal every day. She was re-admitted to our department for the second time to improve her nutritional status, 18 months after the first admission. The results of her ECG (Figure 1), chest X-ray, and blood tests remained normal on her second admission. Her PS had increased to 8, and ibudilast, vitamin C, and coenzyme Q10 were prescribed. During the second hospitalization, she complained of transient chest discomfort lasting 1 to 2 h, which repeatedly occurred at night or early in the morning; negative ST changes in leads II, III, and aVF were observed on ECG (Figure 2), and the serum troponin was negative. Plasma brain natriuretic peptide level was 4 pg/mL, and echocardiography finding was almost normal. She was then referred to a cardiologist for further investigations. Twenty-four-hour holter monitoring showed ST depression with tachycardia, when the patient felt an exacerbation of fatigue from the baseline (Figure 3). A two-step exercise provoked wide ST depression and negative T waves on ECG (Figure 4). A coronary angiogram was not performed, as it was considered too invasive for an emaciated patient; furthermore, the patient did not consent to this procedure. She was diagnosed with vasospastic or microvascular angina and prescribed a calcium channel blocker, in addition to previous Kampo medicines. Although other ME/CFS symptoms remained, the patient was discharged after approximately 1 month of hospitalization, as she gradually showed slight improvement in food intake, as well as decrease in anginal symptoms.

## 3. Discussion

In this report, we presented a case of successfully diagnosed angina masked by ME/CFS deterioration. Endothelial dysfunction was previously correlated to ME/CFS and shown to reflect disease severity [11]. While the pathophysiology of ME/CFS is not yet fully elucidated [12,13], autonomic nervous system dysregulation, immunological disturbance, viral infections, and metabolic changes were proposed as underlying mechanisms of this malady [14,15,16,17]. Indeed, previous studies cited all mechanisms as being responsible for endothelial dysfunction [18,19,20,21]. On the other hand, endothelial damage is also correlated with vasospastic or microvascular angina. Endothelial dysfunction might lead to hyperreactivity of vascular smooth muscle cells via downregulation of vasodilators (e.g., nitric monoxide) and upregulation of vasoconstrictors (e.g., endothelin-1), resulting in coronary spasm [18]. 

Thus, ME/CFS might be associated with an ischemic heart disease, as a malfunctioning endothelium is associated with ME/CFS, and is also closely related to the development of coronary disease [6,7,8]. In fact, a prior study reported that a significantly larger proportion of patients with ME/CFS with angina-like symptoms showed a negative T-wave on 24-h ECG monitoring, compared to patients with the same symptoms but without ME/CFS [9]. Furthermore, any ECG changes are indicative of a higher risk of coronary heart disease and chronic heart failure. Indeed, heart failure accounts for approximately one-fifth of deaths among patients with ME/CFS [7,19].

Notably, in the present case, an ECG change attributed to angina was recorded in concurrence with the recurrence of ME/CFS (which had a 3-year history) and deterioration of PS. Endothelial dysfunction was reported to reduce cerebral blood flow, which is correlated with disease severity [20,21], and results in further fatigue [22]. Therefore, endothelial function might be potentially used to assist the diagnosis of ME/CFS, and serve as an indicator of its severity and the risk of complications [7,11]. The early initiation of treatment is important to improve the prognosis in patients with angina [23].

Additionally, in the present case, exacerbated fatigue was associated with ECG changes, instead of chest discomfort. Although exhaustion is the central symptom of ME/CFS, patients might commonly complain of angina-like symptoms [24]. This suggests that coronary involvement is overlooked in cases of ME/CFS, as the symptoms of ME/CFS might mimic angina, and vice versa. Therefore, physicians should conduct cardiac examinations, including ECG, not only when patients complain of chest pain but also when excessive exhaustion is evident. Such examinations should also be routinely conducted to avoid the potential risk of missing the diagnosis of heart ischemia. 

In conclusion, we experienced an interesting case in which vasospastic or microvascular angina was detected along with concurrent exacerbation of ME/CFS severity. This suggested that cardiac manifestations can appear as a result of deterioration. As the symptoms experienced by patients with ME/CFS might mimic those of angina, diagnosis of the latter might be easily missed. Thus, it is pertinent that cardiac examinations be conducted in such patients to rule out the possibility of heart ischemia.

## Figures and Tables

**Table 1:** Performance status scoring criteria.

PS 0	The patient can perform the usual activities of daily living and social activities without malaise.
PS 1	The patient often feels fatigue.
PS 2	The patient often needs to rest because of general malaise or fatigue.
PS 3	The patient cannot work or perform usual activities for a few days in a month.
PS 4	The patient cannot work or perform usual activities for a few days in a week.
PS 5	The patient cannot work or perform usual activities but can perform light work.
PS 6	The patient needs daily rest but can perform light work on a “good day”.
PS 7	The patient can take care of himself/herself but cannot perform usual duties.
PS 8	The patient needs help to take care of himself/herself.
PS 9	The patient needs to rest the whole day and cannot take care of himself/herself without help.

## Data Availability

The data presented in this study are available on request from the corresponding author.
